# Neglected puerperal inversion of the uterus: ignorance makes acute a chronic form

**Published:** 2012-07-27

**Authors:** Sardha Minakshi, Atri Shivani, Anjum Arshad

**Affiliations:** 1 Department of Obstetrics & Gynaecology/ JR-3, Department of Surgery, Government Medical College, Amritsar, Punjab, India; 2 Department of Pediatrics, Government Medical College, Amritsar, Punjab, India; 3 Department of Obstetrics & Gynaecology, Government Medical College, Amritsar, Punjab, India

**Keywords:** Inversion of uterus, acute puerperal inversion, shock, post partum bleeding, neglect

## Abstract

Inversion of uterus is a rare complication of vaginal delivery. The reported incidence of puerperal inversion varies from approximately 1 in 550 to 1 in several thousand normal deliveries. Maternal mortality has been reported to be as high as 15%, mainly because of associated life threatening blood loss and shock. Early diagnosis, prompt and aggressive management decrease the morbidity and mortality to minimal. We report a case of 21 year old primi, who presented to us with uterine inversion after delivery at a rural set up by untrained birth attendant (“Dai”). She was managed surgically with Haultain's operation and discharged after 5 days. She didn't turn up for follow up and was readmitted after 4 weeks with uterine reinversion associated with endometritis. A recent case is described, followed by a short review of literature.

## Background

Uterine inversion is a rare complication of vaginal delivery which if not recognized and treated immediately, can lead to life-threatening bleeding and shock. The reported incidence of uterine inversion varies considerably in the literature, with reports ranging from approximately 1 in 550 to 1 in several thousand deliveries [1–4]. Maternal mortality has been reported to be as high as 15 % [1–3]. Its presentation can vary from acute to chronic forms. Acute inversion generally occurs during or immediately after childbirth, while chronic form manifests gradually during puerperium.

## Observation

We report a case of 21 year old primipara, who presented in Gynecology Outpatient Department (OPD) of Government Medical College, Amritsar, India with complaints of continuous vaginal bleeding. She had history of normal delivery conducted by an untrained birth attendant, nearly 22 hours back in a rural set up. Initially, she had normal vaginal bleeding with slight abdominal discomfort. Next morning, while passing urine she felt severe abdominal pain and noticed chunks of clots coming through the vagina. She was rushed to a peripheral health center (PHC) in village, from where eventually she was referred to our institute, with the diagnosis of post partum hemorrhage (PPH).

On presentation, she had tachycardia (HR=136) and hypotension (BP=98/64). She was given blood transfusion and her vitals were stabilized. On per-abdomen examination, uterine fundus could not be palpated but cupping was appreciated. Speculum examination revealed fresh bleeding and a mass of 8 X 10 cm inside the vagina, but fundus of the uterus was not felt. A clinical diagnosis of acute inversion of uterus was made. Under anesthesia, vaginal manual reposition was attempted, which failed due to tight cervical ring. Then, O Sullivan's hydrostatic method was tried, which was also unsuccessful. Finally, abdominal reposition was planned.

On opening the abdomen classic flowerpot appearance was visible with cupping of uterus with the tubes and ovaries inside the cupped uterus. After unsuccessful attempt via Huntington's procedure, the posterior ring of inversion was cut vertically and the uterus was pulled up (Haultain procedure). Post operative period was uneventful and the patient was discharged after 5 days.

She didn't turn up for follow up and after 4 weeks she presented with complains of fever, cough, sever pelvic discomfort and continuous vaginal bleeding. She has severe pallor and on local and speculum examination, blood mixed foul smelling discharge and a mass of 5X5 cm was seen. Diagnosis of severe anemia with incomplete reinversion, along with endometritis was made. She was investigated thoroughly, blood transfusion given and culture sensitive antibiotics were started. Reposition of inverted uterus was done vaginally via continuous digital pressure, under oxytocics infusion. Patient was discharged after 10 days and during this period her hospital stay was uneventful.

## Discussion

The puerperal uterine inversion is a rare complication of the third stage of labour. It is defined as the turning of the uterus inside out, usually following child birth. It is classified according to the delay between the delivery and the diagnosis of the uterine inversion as acute, sub-acute and chronic inversion. The acute inversions occur immediately or within 24 hours; sub-acute inversion occurs after 24 hours and before four weeks; and chronic inversion occurs more than four weeks, after the delivery [5]. The prevalence of each class of inversion is 83.4%, 2.62% and 13.9% respectively [6, 7].

The mechanism of the uterine inversion is not completely understood. Some extrinsic factors such as oxytocic arrest after a prolonged labour, umbilical cord traction or abdominal expression have been cited as a cause by few authors [8, 9]. While intrinsic risk factors such as primiparity, pauciparity [1, 5, 7] uterine hypotonia secondary to twin pregnancy, betamimetic, fundic or accrete placenta, fundic myoma and short umbilical cord [7, 10] has also been reported.

Idiopathic inversion is very rare. Usually the clinical features of chronic inversion include chronic vaginal discharge, irregular vaginal bleeding, anemia, and pelvic discomfort [11]. Clinical diagnosis of uterine inversion is difficult unless the fundal depression can be palpated on rectal examination. Sometimes its presence may not be appreciated until the time of surgery [12].

In our case, acute uterine inversion which was managed successfully by abdominal reposition, presented later as re inversion. Probable cause is ignorance, bad compliance, poor socioeconomic status leading to uterine infection and secondary post partum haemorrhage.

Management of uterine inversion should be step-wise, comprising of non-surgical and surgical approaches. The first step is summoning appropriate help, followed by resuscitation and stabilisation of the patient. Once the diagnosis has been established, an immediate attempt should be made to replace the uterus manually through the vagina past the cervical ring, with pressure directed upwards towards the umbilicus. This is commonly referred to as Johnson's method [13]. Should this fail hydrostatic replacement is the next step. This technique was initially described by O'Sullivan in 1945 and was subsequently modified by Ogueh & Ayida in 1997, who employed the use of a 6 cm silastic ventouse cup to correct uterine malposition [14]. Since then, Antonelli et al. have described a new technique of using a silastic cup at the time of laparotomy [15]. Use of an intrauterine Rusch balloon catheter(Modified hydrostatic method) has also been reported [16].

Surgical procedures are indicated when manual reduction fails. Huntington procedure involves a laparotomy to locate the cup of the uterus. Allis forceps is used to gently apply upward traction until the inversion is corrected. Haultain technique involves a longitudinal incision on the posterior cervical ring and reversal by gentle traction as in the Huntington procedure [1, 3]. A modified laparoscopic reduction has also been reported recently [17].

## Conclusion

Uterine inversion is an unusual and potentially life-threatening complication of third stage of labour. This is particularly important in India as approximately 80% of deliveries are conducted by untrained birth attendants (Dai's) and they hardly use any oxytocic agents. The morbidity and mortality associated with this complication can be decreased by prompt diagnosis and early initiation of pertinent treatment. We conclude that proper education and training regarding placental delivery, diagnosis and management of uterine inversion should be given to traditional birth attendants and family physicians, so that this potentially life-threatening condition can be prevented.
